# Bone Diseases: Current Approach and Future Perspectives in Drug Delivery Systems for Bone Targeted Therapeutics

**DOI:** 10.3390/nano10050875

**Published:** 2020-05-01

**Authors:** Giulia Chindamo, Simona Sapino, Elena Peira, Daniela Chirio, Mónica Cristina Gonzalez, Marina Gallarate

**Affiliations:** 1 Department of Drug Science and Technology, University of Turin, 10125 Turin, Italy; giulia.chindamo@unito.it (G.C.); elena.peira@unito.it (E.P.); daniela.chirio@unito.it (D.C.); marina.gallarate@unito.it (M.G.); 2 Instituto de Investigaciones Fisicoquímicas Teóricas y Aplicadas, Facultad de Ciencias Exactas, Universidad Nacional de La Plata, La Plata 1900, Argentina; mcgonzalez.quim@gmail.com

**Keywords:** bone diseases, bone regeneration, nanotechnology, targeted delivery systems, calcium phosphate

## Abstract

Bone diseases include a wide group of skeletal-related disorders that cause mobility limitations and mortality. In some cases, e.g., in osteosarcoma (OS) and metastatic bone cancer, current treatments are not fully effective, mainly due to low patient compliance and to adverse side effects. To overcome these drawbacks, nanotechnology is currently under study as a potential strategy allowing specific drug release kinetics and enhancing bone regeneration. Polymers, ceramics, semiconductors, metals, and self-assembled molecular complexes are some of the most used nanoscale materials, although in most cases their surface properties need to be tuned by chemical or physical reactions. Among all, scaffolds, nanoparticles (NPs), cements, and hydrogels exhibit more advantages than drawbacks when compared to other nanosystems and are therefore the object of several studies. The aim of this review is to provide information about the current therapies of different bone diseases focusing the attention on new discoveries in the field of targeted delivery systems. The authors hope that this paper could help to pursue further directions about bone targeted nanosystems and their application for bone diseases and bone regeneration.

## 1. Introduction

Bone is one of the most important organs of the human body which sustains and protects other organs, produces blood cells, and regulates hormones [1]. It is composed of a matrix that includes proteins such as collagen, proteoglycans, and lipids, and a mineral phase, generally known as hydroxyapatite (HA) Ca_10_(PO_4_)_6_(OH)_2_ [2]. Besides the mineral and protein component, cells, macromolecules, and blood vessels could also be found in bone. Among such cells, some of them can enhance the healthy structure of bone, i.e., osteocytes, implicated in the signal transduction of mechanical stimuli, osteoblasts, delegated to the secretion of collagenous proteins which constitute the bone material, and osteoclasts, that secrete acids and proteases and degrade the mineralized tissue. Furthermore, on the osteoblast surface, the receptor activator of nuclear factor kappa-B ligand (RANKL) is bound, which is a protein molecule that activates osteoclasts and is able to facilitate the communication between osteoclasts and osteoblasts. In the case of RANKL overexpression, a large amount of degenerative bone disease could occur [3].

Bone diseases include a wide group of skeletal-related disorders that cause mobility limitations and mortality. The treatment of bone diseases requires oral and bolus administration of high doses of drug in order to reach effective drug concentrations in the bone tissue. Such high drug concentrations in blood may induce secondary adverse effects in other organs and tissues [4,5,6]. Therefore, there is a critical necessity to optimize the delivery of effective drug concentrations to the bone disorder site without reaching toxic levels in blood. The local administration of drugs in bone tissue diseases and disorders is an area of active research. 

Nanotechnology is an important research area, having one of its important applications in biomedical sciences and, among these, targeted drug delivery is one of the most studied. Nanosystems used as vehicles should observe a particular affinity for bone inorganic material but should also be able to target the site of bone disease and prolong drug release. The material forms used for this aim include a range of nanoscale compounds like polymers, ceramics, semiconductors, metals, and self-assembled molecular complexes. In general, such nanomaterials should be able to deliver drugs, but also different biomolecules which could allow specific release kinetics and biodistribution and enhance bone regeneration. Several advantages are connected to the use of nanometer sized carriers, i.e., high volume to surface ratios which allow efficient drug adsorption, capability of enhancing drug solubility and chemical stability in biological media, ease of surface functionalization to address specific requirements, enhanced transport across cell membrane reducing drug removal from the body, controlled dissolution rates/drug bioavailability, and size similar to natural bone components (e.g., HA).

Furthermore, to optimize nanocarriers for specific functions, the surface properties of nanomaterials could be tuned by physical or chemical adsorption. For example, different proteins can be adsorbed on the surface promoting osteoblasts functions and an increase of surface charges could have a great impact on the transport to the bone [7]. Furthermore, a better ability to bind calcium ions of HA could be obtained in the presence of negatively charged calcium phosphate (CaP) coatings. Also, several nanomaterials like superparamagnetic iron oxide nanoparticles (SPIONs), gold nanoparticles and quantum dots may be detected by either magnetic resonance (MRI), computer tomography, and/or photoluminescence imaging, which allow the monitoring of drug delivery [8]. 

Despite the fact that nanotechnology has been investigated as a potential strategy to reduce side effects related to the therapies [5,9], most investigated systems did not surpass the transition from laboratory research to clinical application. Therefore, only a few formulations have been approved by the U.S. Food and Drug Administration (FDA) and the European Medicines Agency (EMA) for the treatment of bone cancer. The most relevant example is represented by Mepact^®^ (Millennium, Cambridge, MA, USA), a liposomal formulation of non-pegylated mifamurtide (muramyl tripeptide phosphatidyl ethanolamine, MTP-PE) approved by EMA in 2009 for the treatment of osteosarcoma (OS) [10,11]. For the treatment of osteomyelitis, there are five antibiotic-loaded poly(methyl methacrylate) (PMMA) bone cements approved by FDA: Simplex^®^ P (Stryker Howmedica Osteonics, Mahwah, NJ, USA), Palacos^®^ G (Zimmer, Warsaw, IN, USA), SmartSet^®^ GHV and SmartSet^®^ MHV (Depuy Orthopaedics, Inc., Warsaw, IN, USA) and the Prostalac^®^ prosthesis (DePuy Orthopaedics, Inc.). Furthermore, PMMA antibiotic loaded beads products (Septopal^®^) are widely used and available in Europe [12]. CaP cements (CaPCs) were approved by FDA in 1996 for the treatment of maxillofacial/craniofacial defects or in case of vertebroplasty. Three formulations are commercially available: Alpha-BSM^®^ (Etex Corporations, Cambridge, MA, USA), Bone Source^®^ (Striker How medica Osteonics, Rutherford, NJ, USA), and Skeletal repair systems^®^ (SRS) (Norian Corporation, Cupertino, CA, USA) [13].

Overall, the clinical translation of nanomedicines is hindered by the difficulty to assess reproducible manufacturing, characterization and scale-up, and most of all, to ensure in vivo stability and safety. Moreover, disease heterogeneity and clinical trials strategies require further learning, and regulatory rules require an adequate regulatory background [14].

The aim of this review is to summarize the most recent progress of bone targeted drug delivery systems for the treatment of bone diseases like bone metastases, OS, osteoporosis, osteomyelitis, and bone regeneration. In the first part, we introduce some information about the pathogenesis and current therapies of the above-mentioned pathologies, including advantages and disadvantages, while in the second part, new discoveries about targeted delivery systems are discussed. It is thus hoped that the discussion herein presented could allow the pursuit of further directions about bone targeted drug delivery and its application for bone diseases and bone regeneration [1].

## 2. Bone Disease: Pathogenesis and Current Therapies 

### 2.1. Bone Metastases

Bone is a frequent site of metastasis, particularly due to breast and prostate cancer, while it is less common as a site of primary cancer, especially in adults. 

Based on the primary mechanism of interference with normal bone remodeling, bone metastasis can be classified in three types as reported in Table 1.

Bone metastases are the major cause of morbidity, characterized, for example, by severe pain, pathologic fractures, bone marrow aplasia, and hypercalcemia. Therefore, the treatment of metastasized cancers in bone is important for patients in providing prolonged survival rates. In Table 2, the most used chemotherapeutic agents are represented, with a focus on their mechanism of action and common side effects.

Surgery is indicated only in a few cases, such as long bone fractures or nerve compression, while stereotactic surgery is considered an emergent treatment for the metastases of vertebral bodies and the spinal cord. This therapy shows several advantages (no needs to irradiate large segment of the column and rapidity of the treatment), but currently, there is a low quality of literature, there are no randomized controlled studies, and it is more expensive than conventional radiotherapy [23]. For these reasons, it is necessary to find drug delivery systems to reduce side effects and to increase patient compliance. Furthermore, to obtain a system which can be systemically administered and reach the bone mineralized tissue, it is necessary to focus on the dimension of the carrier because it has to cross the blood-bone marrow barrier, including the clefts of bone marrow sinusoidal capillaries which have a diameter of about 80–100 nm [24].

### 2.2. Osteosarcoma

OS is the commonest bone sarcoma, characterized by the production of osteoid and bone by malignant spindle cells, which affects primarily 10–20 year-old patients. This type of tumor develops mainly in the metaphyseal regions of the distal femur, proximal tibia and proximal humerus, although it can origin in any bone. An OS grows in a radial manner, forming a ball-like mass. Then, it penetrates the bony cortex and it compresses the surrounding muscles, forming a pseudocapsular layer called the “reactive zone” in which nodules (also known as “satellites”) may also arise which are microextensions of the primary mass. Patients typically complain a dull and aching pain that could last for several months and suddenly becomes more severe probably due to tumor penetration of cortical bone or to pathologic fracture [25]. 

Based on its histologic, radiographic, and clinical features, OS can be classified in two groups: intramedullary and cortex-associate (surface) subtypes as summarized in Table 3. 

Currently, chemotherapy is essential in the treatment of OS, because it can decrease the size of the primary tumor through the reduction of its neovascularization and of the number of pulmonary metastasis or delay their appearance. Common treatments consist in a combination of therapies that include preoperative chemotherapy followed by the surgical resection of the primary tumor plus the addition of postoperative chemotherapy. Preoperative chemotherapy leads to necrosis of the primary tumor and is a first treatment of micrometastasis in order to facilitate surgical resection.

Drugs used in this step are doxorubicin (DOX), cisplatin, ifosfamide (also in combination with mesna that prevents hemorrhagic cystitis) and high dose methotrexate with leucovorin as calcium rescue used to prevent myelosuppression and mucositis [26]. The combination of different drugs could avoid chemoresistance and increase the degree of tumor necrosis. In Table 4 [25], the mechanism of action and the side effects of the different chemotherapeutic agents are reported. 

Surgical resection is performed 3–4 weeks after the last dose of preoperative chemotherapy, while postoperative chemotherapy begins 2 weeks later when the tumor necrosis is found to be ≥90% at the time of surgery. In some cases, radiation therapy is used in the treatment of OS, but is only palliative because this type of tumor is highly resistant to radiation.

For this reason, the aim of current research is to improve and develop more target-selective treatments with the aim of enhancing overall long-term survival and reducing side effects.

### 2.3. Osteoporosis

Osteoporosis is a common bone disease in which low bone mass and microarchitectural deterioration of bone tissue are frequent characteristics with an increment of bone fragility and susceptibility to fracture. It is often underdiagnosed and not treated with disease-specific medication. Potential risks for secondary osteoporosis are endocrine disorders, hypogonadism, inflammatory disease, bone marrow disorders, immobilization, malabsorption, low body weight, defective synthesis of connective tissue, or regular intake of drugs with side effects on bone metabolism [27]. Current therapies provide for the interruption or the slowdown of the increased resorptive activity in order to prevent further deterioration. Nitrogen-containing BPs (N-BPs) and the human monoclonal antibody denosumab are very effective in slowing this bone decay. Despite their common end results, there are important differences between these two classes of antiresorptives, such as mode of action, rebound bone resorption, and adverse event profiles (Table 5) [28].

Newer antiresorptives include cathepsin K inhibitors, which act on the cysteine protease cathepsin K, highly expressed in osteoclasts and essential for the digestion of cartilage and bone collagenous matrix. Additionally, another class used in osteoporosis therapy is represented by selective estrogen receptor modulators (SERMs) and tissue-selective estrogen complexes (TSECs).

In Table 6, the most representative drugs of these classes are discussed. 

Nevertheless, antiresorptives are helpful only in bone-forming, but the faulty structure remains. Currently, parathyroid hormone (PTH) is the only available treatment with the potential to reconstruct skeleton. In fact, PTH is capable of stimulating both bone formation and resorption. An alternative to PTH administration is represented by calcium-sensing receptor antagonists (also called calcilytics) which can stimulate the secretion of PTH from the parathyroid glands. Different studies report that these compounds act by reproducing the hypersecretion pattern of the hyperparathyroid state instead of that of pulsatile waves of circulating PTH required for osteoanabolics effects [29]. Another class of drugs attracting the attention of researchers is represented by sclerostin inhibitors. Sclerostin is an antagonist of Wnt signaling and is an important negative regulator of bone formation. It is produced by the gene SOST, and it resulted that sclerostin gene knock-out mice have increased bone formation and strength. The main characteristics of romosozumab and blosozumab, two sclerostin inhibitors currently under investigation, are reported in Table 7.

Strontium ranelate has been promoted as a bone agent of dual action mode, though histomorphometric data showed no significant anabolic action and limited effects on bone resorption. For this reason, the use of strontium ranelate in Europe is limited to the treatment of severe osteoporosis in patients without a history of cardiovascular disease [28].

### 2.4. Osteomyelitis

Osteomyelitis is a bone infection/inflammation, particularly common among the elderly, diabetics, children and people from the Third World Countries. It is caused by pyogenic bacteria living in healthy oral flora, but in some cases, it is also due to fungi infection. For example, *Haemophilus influenzae*, *Brucella suis*, *Mycobacterium tuberculosis*, *Mycobacterium ulcerans*, and *Fusobacterium nucleatum* are some of the commonest pathogens. Among all, staphylococcal infections, mostly caused by *Staphylococcus aureus* and *Staphylococcus epidermidis*, are the major cause of osteomyelitis. Then, a large number of factors, like bacteria, pH change, local edema, vascular obstruction, and leukocyte collagenase, contribute to disease progression. Osteomyelitis is largely widespread in the facial skeleton because it is more accessible to external pathogens and microorganisms. It is also a complication that could occur after orthopedic, maxillofacial surgeries and dental extractions. Timely treatment is required to prevent its diffusion to other sites. Common therapies include the administration of antibiotics by endovenous route until 2–6 weeks (followed by a 6-month course of oral antibiotics when there is a chronic infection) and surgical removal of necrotic bone. Several downsides are related to the conventional therapeutic approach, e.g., side effects due to antibiotic systemic administration and low concentration of the therapeutic agent around the site of infection, which could induce resistance and bone loss that often requires the insertion of implants or prostheses. A widely used option for the local drug delivery of antibiotics is represented by PMMA cements or beads loaded with hydrophilic drugs such as gentamicin sulfate (GS), ceftriaxone, tobramycin (TOB), and vancomycin, although numerous clinical drawbacks like non-biodegradability, need to be surgical removed and exhibition of a burst and inconsistent release profile are connected with their use. For these reasons, it is important to develop locally targeted and minimally invasive therapies for the treatment of osteomyelitis [30,31,32].

### 2.5. Bone Regeneration

Bone possesses the ability to regenerate itself in response to injury, requiring some of biological events of bone induction and conduction which involve a large amount of different cell types and intracellular and extracellular molecular-signaling pathways. Many studies have also confirmed that several growth factors may regulate different phases of bone fracture repair. Among these, the most important are the bone morphogenetic protein (BMP), the transforming growth factor-β (TGF-β), the fibroblast growth factor (FGF), the vascular endothelial growth factor (VEGF), the platelet-derived growth factor (PDGF), the insulin-like growth factor (IGF), the interleukin-1 (IL-1), and the tumor necrosis factor (TNF) [33]. Generally, most bone injuries (in particular fractures) can heal without the formation of scar tissue, restoring the pre-existing properties of the bone.

However, there are different cases, e.g., orthopedic and oral/maxillofacial surgery, in which bone regeneration is very important, or cases in which the regenerative process is compromised, such as in vascular necrosis and osteoporosis.

Currently, several treatment methods are available for previously discussed cases, which could be used alone or in combination. Standard regimens include distraction osteogenesis and bone transport or the use of autologous bone grafts, allografts, bone-graft substitutes or growth factors. In particular, during distraction osteogenesis and bone transport, bone regeneration is induced between the gradually distracted osseous surfaces by the use of external fixators and the Ilizarov technique [34], which provides a versatile fixation by the combination of unreamed intramedullary nails and external monorail distraction devices, or intramedullary lengthening devices. In this way, during distraction, regenerate bone arises between the entire cross-sections of each distracted bone surface [35]. In any case, these methods are technically difficult and exhibit several drawbacks. Bone grafting, and in particular autologous bone, is a common surgical procedure because it brings together all properties desired in a bone graft material, i.e., osteoinduction, osteogenesis, and osteoconduction. This method offers several advantages like histocompatibility and non-immunogenicity which are able to reduce to a minimum immunoreactions and transmission of infections, but at the same time there are some complications that could represent a discomfort for the patient. An alternative is represented by allogenic bone grafting, obtained from human cadavers or living donors, which could bypass the problems of the previous method but, at the same time, exhibits low osteoinductive properties, issues of immunogenicity, rejection reactions and the possibility of infection transmission. Bone-graft substitutes are other alternatives to autologous and allogenic bone grafts, which consist of a scaffold made of synthetic or natural biomaterials, like collagen and its composites [36], HA, CaPCs, and glass ceramics, able to promote bone regeneration [37]. The ideal materials for bone fracture repair should have some characteristics, such as good biocompatibility, appropriate biodegradability, good mechanical properties, osteoinductivity, and osteoconductivity. A 3D-porous structure and the possibility to be easily sterilized should be desirable. Examples of currently used materials for bone fracture repair also include bone, bone cements, metal, ceramic, and polymer. All of them exhibit advantages and disadvantages, some of which are depicted in Table 8 [33].

## 3. Drug Delivery Systems for Bone Diseases

Among the broad spectrum of nanotechnology-based drug delivery, several systems are used for the treatment of bone disease. Here, we propose a brief overview of the commonest types of nanosystems exploited for this aim.

- Extracellular vesicles (EVs): proposed for their ability to load proteins and nucleic acid in various fields such as oncology and regenerative medicine [38,39].
- Liposomes: spherical vesicles with an aqueous core surrounded by a single or multiple bilayer membrane, proven to be suitable tools also in several bone diseases [40].
- Microspheres: generally made of natural, semisynthetic, and synthetic polymers, frequently applied in anticancer therapy [41,42].
- NPs: solid, colloidal particles of different materials which can be functionalized or coated to improve stability and receptor binding [43].
- Beads: frequently used as bone drug carriers, generally based on alginate, chitosan, gelatin and cellulose [44,45].
- Dendrimers: nanosized synthetic polymeric macromolecules composed of a central core with branching units that can provide cytotoxicity [46,47].
- Conjugates: especially polymer-drug [48] and lipid-drug types, exploited in bone diseases [49].
- Cements: obtained by mixing solid and liquid parts until a viscous paste is formed, used both as bone filler during surgery and as drug carrier [2,50,51].
- Ceramics: inorganic non-metallic solids obtained by heating [52], including β-tricalcium phosphate (TCP), HA, and mesoporous silica [53].
- Nanodiamonds: carbon-based nanomaterials which exhibit a large surface area and could be functionalized to conjugate various compounds or drugs, suitable as photoluminescent probes [54].
- Nanocrystals: usually stabilized by surfactants and/or polymers to prolong the circulating time in the blood and facilitating tumor accumulation [55].
- Nanohybrids: which combine the mechanical and thermal stability of the inorganic component and the ease of processability of the organic one (e.g., clay-based, carbon-based or metal/metal oxide based nanohybrids) [56,57].
- Polymeric gels: solid–liquid systems with polymeric matrix broadly crosslinked forming a 3D network of particular interests for biomedical applications [58].
- Matrices: consisting in homogenous dispersions of solid drug in a polymer mixture [59].
- Nanocapsules: systems with a liquid/solid core with a protective coating which delays the release of the entrapped active compounds [60].
- Implants: man-made materials (metals, polymers or ceramics) embedded in the human body to repair or replace damaged tissues [61,62].
- Scaffolds: biocompatible templates which are similar to a platform and promote the attachment and the growth of cells, thus used for tissue regeneration and surgical repairs [63].
- Nanoplatforms: biocompatible and biodegradable engineered systems based on NPs, liposomes, micelles, and dendrimers, capable to load both hydrophilic and lipophilic drugs and molecules, making them attractive systems mainly in cancer therapy [64].

In the following paragraphs, we propose a focus on the above-mentioned drug delivery systems organized according to main bone diseases. It is noteworthy that, to date, clinically approved nanotechnology-based therapeutic agents concern those known as “first generation agents” which are composed by liposomes or polymers, with no active targeting, and containing only one type of drug.

### 3.1. Bone Metastases

In the last decades, different drug delivery systems have been developed for bone metastases. Many attempts have been centered on NP drug delivery systems, like HA NPs, liposomes, and micelles, with the aim of increasing their permeability to the blood–bone marrow barrier and the retention time. For example, in a recent study (2017), some researchers developed water-in-oil (W/O) microemulsion in which a cisplatin prodrug (DSP) was included in polymer nanoparticles (CPNs) functionalized with alendronate (ALN) to obtain a selective accumulation in the bone metastatic lesion and a prolonged drug circulation time [65].

Another type of polymeric NPs (PNs) with high bone affinity were prepared by Hirano et al. (2017) using poly(ethylene sodium phosphate) (PEP_n_ Na). These systems show low cytotoxicity with high colloidal stability (at least 1 week) [66].

In 2017, Chu et al. designed a novel bone-targeting NP combining the anti-osteoporosis property of BPs and the similarity of CaP sediments to HA. In this study ALN was conjugated to PEG 2000 (ALN-PEG2000-ALN) and then anchored to the surface of CaP NPs with the aim to prolong the circulation time and obtain a selective delivery to the bone [67].

Another research carried out by Chaudhari et al. (2012) involved the development of zoledronate (ZOL) anchored PLGA NPs loaded with docetaxel (DTX) in order to combine the cytotoxic effects of both DTX and ZOL. In vitro studies showed enhanced activity. Moreover, in vivo animal study using technetium-99m radiolabeling indicated prolonged blood circulation half-life, less liver uptake, and a higher retention of ZOL targeted NPs at bone site [68].

Further, different studies suggest that osteocytes (which secrete HA) are also involved in the development of cancer bone metastasis. It is possible that these cells are able to control the activity of bone metastasis and osteoclastogenesis through the expression of some growth factors (e.g., RANKL). So, the inhibition of osteocyte-mediated osteolysis represents a probably better approach for bone tumor treatment. For this reason, Qiao et al. (2017) developed ZOL-anchored bimodal mesoporous silica covered gadolinium (III) NPs loaded with plumbagin (PL, a medicinal plant-derived naphtoquinone with anticancer properties [69]) to detect and treat bone metastasis at an early stage. Results demonstrated that developed NPs could regulate osteoclastogenesis, and they could also be applied for other bone related disease, like osteoporosis and osteoarthritis, in order to achieve theranostic effects [70].

Another interesting approach is focused on peptides with aspartic acid (ASP) repeating units for bone-targeting delivery. These peptides are negatively charged and able to bind calcium ions on the surface of HA. Additionally, amino acids exhibit a lower rate of cytotoxicity because they can be degraded by enzymes and cleared from the body after the binding with HA. For this reason, Ke et al. (2017) developed a dual-targeting liposomal system with Asp8 and folate that could target tumor cells in the bone. [71].

Another research carried out by Anada et al. (2009) was based on the development of DOX-loaded CaP-binding liposomes. They synthesized an amphipathic molecule bearing a BP head group with high affinity and selectivity for HA. The results showed a reduction of the number of tumor cells with above-mentioned systems; for this reason, they could be used for different bone-related diseases (e.g., osteoporosis, rheumatoid arthritis, and multiple myeloma) and loaded with many drugs [72].

**Figure 1:**
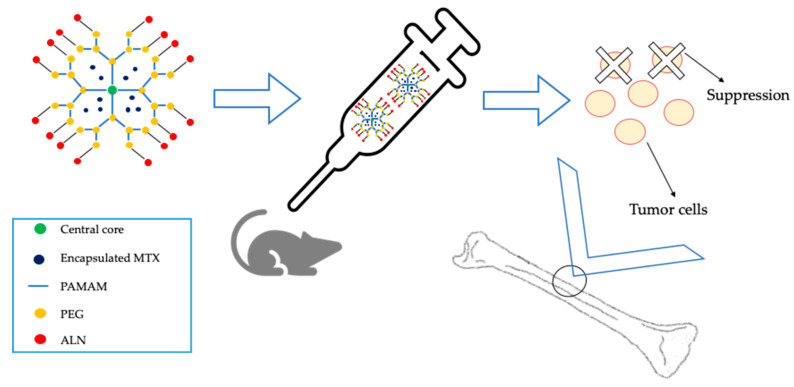
Schematic representation of methotrexate (MTX)-encapsulated dendrimers for the treatment of bone metastasis which allow the suppression of tumor cells in mice.

Dendrimers are other promising drug carriers. Yamashita et al. in a recent study (2018) developed bone-targeting dendrimers in which ALN was covalently linked to a PEG-conjugated polyamidoamine (PAMAM) dendrimer (PEG-PAMAM-ALN) for bone targeting of MTX. After intravenous injection in mice, MTX-loaded PEG-PAMAM-ALN were effectively distributed to the bones and suppressed the number of tumor cells localized in the bone (Figure 1) [73].

Previously, Clementi et al. (2011) investigated a pegylated dendrimer glutamic acid (GLU) (H_2_N-PEG-β-GLU-(β-GLU)_2_-(COOH)_4_) coupled with paclitaxel (PTX) and ALN with the aim of obtaining a better targeting and enhanced activity [74]. Results reported that PTX-PEG-ALN were nontoxic and showed an IC50 similar to free PTX with increased tumor cell killing efficacy [75].

Another tool to treat bone metastasis is represented by drug-conjugates, a class of carriers which can protect drugs from too rapid degradation and interactions with other drugs or with the biological environment and enhance the absorption of the drugs into the tissues. In 2015, Ye et al. synthesized ALN-monoethyl adipate-(hydrazine)-DOX conjugate (ALN-MA-hydrazine-DOX) to deliver DOX to the bone. The results showed a high absorption capacity of the conjugate and a high release rate in the 5.0–6.0 pH range [76]. Also, ALN-MA-hydrazine-DOX conjugate exhibited high cytotoxicity on wild-type and DOX-resistant tumor cells, without systemic toxicity [77].

In 2011, Zheng et al. conjugated catalase with 3,5-di(ethylamino-2,2-bisphosphono) benzoic acid (Bip) (catalase-Bip), that is a derivate of BPs, with PEG (PEG-catalase) or with both Bip and PEG (PEG-catalase-Bip). Catalase was chosen because it is an enzyme involved in the degradation of hydrogen peroxide and other reactive oxygen species (ROS) which play an important role in the development of tumor metastasis, osteoclast activation, and osteolysis [78]. For this reason, through chemical modification, it could be used as therapeutic agent in bone diseases. At the same time, PEG-conjugation could increase the retention time of catalase and reduce the uptake of the enzyme by liver [79].

In 2004, El-Mabhouh et al. focused their attention on the synthesis of two types of BPs conjugates with 5-fluorouracil as a chemotherapy agent and with diethylenetriaminepentaacetic acid (DTPA) as a carrier of cytotoxic radionuclide. Thereby, ^99m^Tc-labeled BPs conjugates with DTPA and 5-fluorouracil showed a rapid clearance from the blood and many other tissues, but a substantial retention of the BPs in bone [80].

Nanoplatform and cement are less common systems, although they can be considered as alternative tools for the treatment of bone metastasis. Sun et al., in a recent work (2019), developed a nanoplatform with gold nanorods enclosed inside mesoporous silica NPs (Au@MSNPs) and conjugated with ZOL. In this project, researches decided to add the gold nanorods to the system, as photothermal agents, because different studies suggest that photothermal therapy (PTT) is effective in order to ablate several types of cancers, under near-infrared (NIR) laser irradiation. Moreover, hyperthermia could promote the efficiency of chemotherapy [81]. Au@MSNPs-ZOL showed promising results like good bone-targeting capacity and ability to inhibit osteoclast differentiation while promoting osteoblast one. Furthermore, the combination with NIR could inhibit tumor growth, inducing cancer cell apoptosis even if further studies need to consider the overall bone metabolism balance [82]. In 2011, Lopez-Heredia et al. developed a local drug delivery system, carried with chemotherapeutic agents using CaPCs, which are biocompatible materials, frequently used for bone filling applications and with fewer-invasive surgical procedures. Furthermore, CaPCs, compared with PMMA bone cements, exhibit better properties, e.g., crystalline affinity to bone tissue and the possibility to be resorbed and replaced with bone tissue in vivo. PTX was chosen as chemotherapeutic agent and was loaded onto CaPC discs by absorption. From the results, it appeared that the slow and gradual release of PTX from CaPCs was effective against MDA and OST cells. Full drug release was expected to be reached after cement degradation, providing a prolonged local delivery effective against the formation of secondary metastasis [83].

Concluding, we can assume that nanomedicine has developed several therapeutic modalities for bone metastases. Among the several NPs and liposomes and dendrimers described in literature, only some of them have been used in preclinical animal models showing increased prolonged circulation times and bone tissue affinity with consequent increased drug efficacy and decreased toxic side effects, whilst most experimental results consisted in in vitro studies.

Bone cements, even if already used in surgical implants, are new and interesting tools that are currently evaluated to be exploited for drug delivery, but further attention should be given to evaluate their safety and effectiveness.

### 3.2. Osteosarcoma

Recent studies have involved the use of EVs and in particular exosomes, as potential drug delivery systems to the bone. It seems that EVs secreted by tumor cells are involved in cancer development, survival, and drug resistance, but many researchers try to use them as cancer-specific drug delivery or to detect cancer metastasis [84]. In 2019, Abello et al. labeled exosomes obtained from mesenchymal stromal cells (MSCs) with gadolinium or with NIR fluorescent dyes, demonstrating that MSC exosomes actually accumulate in OS tumors, inhibiting human and mouse OS cell proliferation in vitro in a dose-dependent way [85]. Moreover, due to the host defense properties of exosomes, Rayamajhi et al. (2019) developed small EVs hybridized with liposomes, which due to their colloidal stability, drug loading and pH-sensitive sustained drug release resulted to be a valid tool in tumor targeted drug delivery [38]. Another research carried out by Qi et al. (2016) involved the development of a dual-functional exosome-based superparamagnetic NP cluster. Data exhibit an enhanced cancer targeting and tumor growth suppression of drug-loaded exosomes under an external magnetic field, suggesting that the incorporation of magnetism in exosomes could expand their biomedical application [86].

Novel formulation of chemotherapeutics also involved NPs, developed with the aim of optimizing the pharmacokinetic profile, improving drug targeting specificity and reducing toxicity with the maintenance of the efficacy [87]. In this perspective, in a recent study, Wu et al. (2017) functionalized HA NPs with medronate (as a bone-targeting moiety) and JQ1, (a chemotherapeutic agent). Data showed that JQ1-loaded HA NPs exhibit an interesting selectivity and prolonged drug release, being more toxic to tumor cells rather than to primary fibroblasts, thanks to the presence of medronate that selectively attacks and destroys cancer cells without negative effects on healthy fibroblasts with an inhibition of OS cell migration from the tumor spheroids. [88].

With the aim of developing a local delivery of cisplatin to malignant solid tumors and to reduce its toxic effects, in 2004, Barroug et al. developed a system based on NPs made of different type of apatitic CaP. A better activity of the samples was obtained when cisplatin was adsorbed on carbonated apatite and poorly crystallized HA with reduced side effects like loss of weight and kidney damage [89].

For the treatment of OS, liposomes are other drug delivery systems attracting the interest of researchers which are frequently used to encapsulate DOX in order to obtain a target delivery and reduce its toxic effects like cardiotoxicity and congestive heart failure. To deliver DOX in a specific way to OS cells, it is necessary that liposomes could target to cell with surface receptors specific for tumor cells but not for healthy ones. Previous studies found that a type of tyrosine kinases receptor, called ephrin Alpha 2 surface receptor (EphA2) is highly upregulated on OS cells. The interaction between EphA2 and its ligand (ephrinA1) leads to intracellular events such as proliferation, migration and cell survival, that are important in tumorigenesis. For this reason, Haghiralsadat et al. (2017) conjugated DOX-loaded liposomes with YSA, a 12-amino acid peptide, obtaining that the system YSA-liposomes-DOX could be efficiently targeted to the human SaOs-2 OS cell line and that 31 days are required before the complete release of DOX was reached, making these systems a potetial tool for other cancers presenting an overexpression of EphA2 [90]. At a later stage (2018), to overcome OS multidrug resistance, Haghiralsadat et al. used siRNA to target intracellular kinase MAPK8IP1 (JNK-interacting protein 1 [JIP1]) founding that JIP1 siRNA could restore chemosensitivity to DOX. At the same time, the resulting system is stable with a high loading efficiency of DOX and siRNA and shows strong cytotoxic effects OS cells but not to primary bone cells [91].

Another type of drug delivery system that could be useful in OS therapy is represented by microspheres, which could be used as drug carrier matrices. In 2018, Liu et al. prepared CaP porous microspheres loaded with DOX, which exhibit high drug-loading capability and an enhancement in the inhibition of the proliferation of OS cells in vitro with a promotion of the enrichment of DOX in OS cells. Furthermore, a great potential for OS drug delivery and osteogenic differentiation in bone regeneration could be observed [92]. With the same purpose, Zhou et al. (2017) developed calcium phosphate-phosphorylated adenosine (CPPA) hybrid microspheres to exploit the influence of adenosine on bone metabolism and on bone cells differentiation of bone marrow cells. They obtained a system with a high DOX-loading capacity and pH-sensitive drug release properties with an enhancement of in vitro/in vivo therapeutic efficiency for different OS cell lines and subcutaneous tumors in nude mice [93].

In some studies, CaPCs, used as potential carrier for therapeutic agents in orthopedic application, revealed a sustained-release capacity higher than other bone cements. In 2011, Tanzawa et al. developed CaPCs containing the association of cisplatin plus caffeine which seemed to be effective in reducing bone tumor growth probably by a sustained release of the drug and a mechanism of inhibition of DNA repair [94].

Tani et al. (2006) investigated the feasibility of the use of DOX-loaded CaPCs as a postoperative filler material able to release the antitumoral drug. It was found that the presence of DOX doesn’t influence CaPCs strengths, although bone formation around the DOX-loaded CaPCs is slower than CaPCs alone. Also, DOX effects can last longer, (until 15 days), but only DOX particles located on the surface of CaPCs can be released, suggesting useful the induction of CaPCs degradation to maintain the local concentration of DOX for a longer time [95].

Another two types of systems are described in literature: β-TCP beads and nanohybrids. In 2017 Hess et al. prepared β-TCP beads loaded with cisplatin and DOX and then incorporated in a HA matrix scaffold. It seems that the co-delivery of cisplatin and DOX may yield strong synergy in the efficacy with a higher response rate, as suggested by results which show a short cisplatin burst release, while DOX release can last for up to 40 days. Moreover, the incorporation in the matrix could reduce cytotoxicity of the co-loaded composites [96].

Gonçalves et al. (2013) developed Laponite^®^/DOX/alginate nanohybrids that are capable to sustain drug delivery and exert a prolonged anticancer activity. DOX was loaded into pH-sensitive biocompatible anionic Laponite^®^ nanodisks coated with alginate to prevent drug burst release. The results demonstrate several advantages of formulated systems like high entrapment efficiency (80.8 ± 10.6%), pH-sensitivity, biocompatibility, and a sustained drug release behavior. Also, Laponite^®^/DOX/alginate nanohybrids can be internalized by OS cell line (CAL-72) and show a noticeable cytotoxicity to cancer cells higher than free DOX, making these nanohybrids excellent platforms for drug delivery [97].

**Figure 2:**
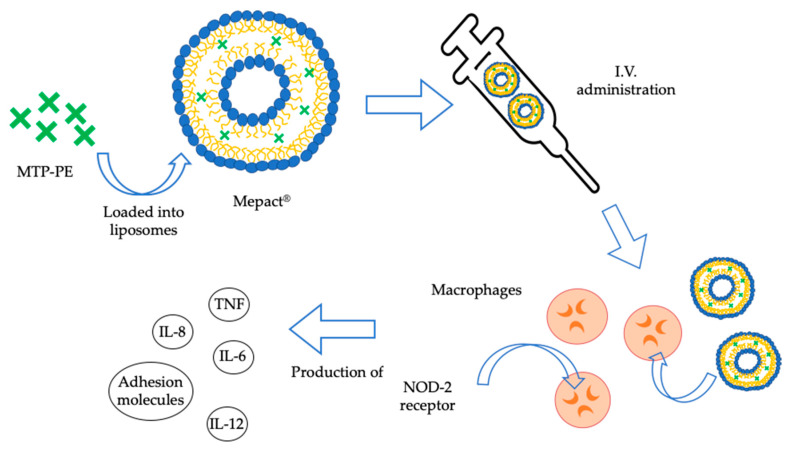
Mechanism of action of liposomal muramyl tripeptide phosphatidyl ethanolamine (MTP-PE). It is a specific ligand of NOD2 which is an intracellular receptor in monocytes, dendritic cells and macrophages: the activation of these cells produces cytokines e.g., tumor necrosis factor (TNF), several interleukins (IL), e.g., IL-6, IL-8, IL-12 and adhesion molecules without effects on tumorigenic cells.

Notably, the various drug delivery nanocarriers designed for OS treatment, so far discussed, are still at experimental stage and further studies are needed before clinical application. Certainly, among all, a significant role seems to be played by CaP NPs or CaPCs/CaP beads or β-TCP nanoderivatives, which are considered as interesting nanocarriers to bone tissues thanks to their biocompatibility and their accumulation capacity in bone tissues. Nevertheless, as previously reported, currently the only nanosystem approved by EMA for the treatment of OS is represented by liposomal non-pegylated MTP-PE (Mepact^®^, Millennium, Cambridge, MA USA). Guidelines provide for the administration through intravenous (I.V.) infusion over a period of 1 h. In Figure 2 the mechanism of action of MTP-PE is shown. In vitro tests performed on human monocytes led to the death of allogenic and autologous tumor cells, while in vivo experiments in murine models resulted in the inhibition of tumor growth [98].

### 3.3. Osteoporosis

Researchers have currently focused their attention on the development of innovative drug delivery systems which can increase the drug access to the bones and obtain a controlled drug release in order to maintain required drug concentration levels for a long period of time without reaching toxic level or dropping below the minimum effective level [99]. Many studies concern the development of CaPCs loaded with BPs in order to allow a local release of the drug which might effectively increase bone implant stability. With this aim, Gong et al. designed CaP silicate cements (CaPSC) and calcium silicate cements (CSC) loaded with risedronate (RA) to obtain biocompatible systems able to promote osteoblast proliferation and to reduce the prevalence of osteoporosis in animal models [100,101]. Similarly, Jindong et al. developed CaPCs with different concentrations of ALN which exhibited a drug release up to 21 days and a good biocompatibility [102,103]. Some studies also reported the use of BMP-2 to enhance osteoinduction in bone substitutes, since the available combination of BMP-2 with CaPCs provides a slow release rate of the factor and has a long degradation period. For this reason, in 2010, Li et al. developed a composite bone substitute with CaPCs with BMP-2-loaded gelatin microspheres (BMP-2/GM/CaPCs). Results suggest that BMP-2/GM/CaPCs composite can release more rhBPM-2 than the system without gelatin microspheres and it can accelerate the bone healing of osteoporosis in vivo. Furthermore, BMP-2/GM/CaPCs provides a better bone mineralization after 45 days of implantation [104]. A problem which can occur during alloarthroplasty is represented by infections. To overcome these troubles and try to fill the missing regions of bone and provide mechanical strength, Tanaka et al. (2002) created a CaPC containing arbekacin sulfate and polylactic acid (PLA) as an implant for the release of antibiotics. Data confirmed the hypothesis of this study, for this reason, developed systems could be considered by the researches useful tools in bone filling and stiffening [105].

Implants can be considered as an alternative drug delivery system in osteoporosis treatment. A study carried out in 2018 by Liu et al. reported that biomimetically deposited CaP-based coatings of prostheses could be considered as a vehicle for the targeted delivery of some growth factors. For this reason, they investigated the release mechanism and the kinetics of a CaP-based implant-coatings loaded with BMP-2 by use of a radiolabeled derivate (^131^I-BMP-2). The release of BMP-2 from the depot appears to be characterized by a burst-release kinetic profile [106]. In 2014, Pyo et al. adopted a biomimetic method to coat titanium implants with CaP incorporating different concentrations of ZOL (8, 80, 800 µg/mL) to promote peri-implant bone formation in osteoporotic cases. The 800 µg/mL treatment group exhibited the best results, showing bone around implants which were more similar to typical plate-like trabecular bone and with ideal trabecular bone structure [107]. In 2005, Peter et al. demonstrated the effectiveness of the use of a local BPs delivery (ZOL) from a CaP coating to increase the mechanical fixation of an orthopedic implant. It resulted that the increase in peri-implant bone density was ZOL concentration-dependent and that the ZOL release positively influenced the structure of bone and, probably, the mechanical stability of the implant [108].

To reduce side effects connected with the systemic use of BPs in the treatment of osteoporosis, different studies were focused on the development of CaP composite materials. For example, Forte et al. (2017) functionalized octacalcium phosphate (OCP) with ALN and ZOL in order to obtain a good compound suitable for local administration. Both drugs stabilize OCP by preventing the hydrolysis, but better results of bone cells viability and interaction with OCP structure were obtained with ZOL. Both ZOL and ALN maintained their antiresorptive effects even when incorporated in OCP [109].

Some studies involved the use of CaP NPs as a potential carrier for the treatment of osteoporosis. In 2013 Chen et al. developed trace elements (TEs)-codoped CaP particles and examined TE retention and bone consolidation in osteoporotic animal models. In fact, it seems that TEs play a critical role in the maintenance of hard tissue function due to their essential and nutritional effects [110]. Pareta et al. (2008) developed iron oxide NPs evaluating their cytocompatibility with osteoblasts. Directing such NPs to bone disease under an external magnetic field (MRI) could promote site-specific bone growth. At a later stage, iron oxide NPs were coated with CaP in the presence of bovine serum albumin (BSA) and citric acid (CA) to reduce particle aggregation and increase osteoblast density after one day [111].

Sometimes, scaffold could be considered as a valid alternative approach for the treatment of osteoporosis. For example, the studies of Chou et al. (2016) were based on the implantation of Foraminifera exoskeletons hydrothermally converted to β-TCP scaffold material for the delivery of simvastatin which has been shown to promote bone regeneration. From their results it appeared that β-TCP with or without simvastatin is able to induce bone formation in osteoporotic mice and that a slower release of simvastatin could be obtained with drug-loaded scaffolds. However, further studies are required to find the optimal concentration to induce therapeutic effects [112]. In 2015, Liporace et al. observed the dose-dependent effect of local delivery of rhBMP-2 using a HA/TCP carrier. Two different doses of rhBMP-2 were tested, exhibiting significantly higher bone mineral density than controls. After eight weeks, better results were obtained with the high dose. Biomechanical tests revealed dose-dependent trends in all samples with minimal significant differences [113].

Lastly, some articles found in literature reported the use of other drug carriers like nanodiamonds, CaP nanocapsules, CaP matrix, microspheres and auto-forming gel as alternative tools in the treatment of osteoporosis. Ryu et al. (2016) conjugated ALN onto a nanodiamond surface to obtain a synergistic effect. Developed systems show a specific uptake and accumulation to bone tissue with an increment of bone mechanical strength [114]. Ito et al. (2012) developed CaP-coated deoxycholate micelles including simvastatin to obtain a delayed release of simvastatin which could reduce its side effects. Actually, it appears that CaP encapsulation reduced the cytotoxicity in cultured cells and suppressed inflammation leading to a whole-body therapeutic effect. For this reason, such biocompatible CaP nanocapsule is expected to be a novel tool for the delivery of hydrophobic drugs [115].

**Figure 3:**
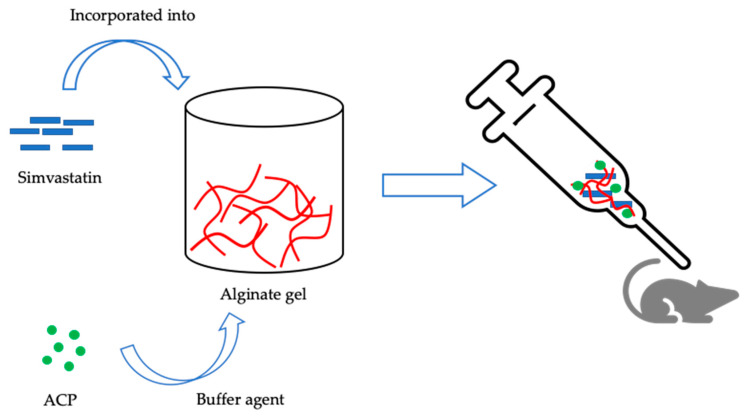
Scheme representing the preparation of simvastatin-loaded alginate gel which becomes a soft carrier after injection in mice. Amorphous calcium phosphate (ACP) acts like a buffer to avoid the acidification of the gel-environment prolonging drug release.

In another study carried out by Ito et al. (2012), simvastatin was incorporated in an alginate gel where highly amorphous calcium phosphate (ACP) was added as a buffering agent to avoid the acidification of the gel-environment (Figure 3).

When injected in osteoporosis rat, a soft gel was formed in the injected site, providing a continuous release of simvastatin under the acidic condition and exhibited high therapeutic effect on the osteoporotic rat demonstrating that auto-forming alginate gel represents a novel controlling device for the delivery of simvastatin [116]. In 2010, Kim et al. developed CaP microspheres which were loaded with ALN during microsphere formation, providing a better drug loading and a simpler fabrication process. Preliminary in vitro results show the potential of bioabsorbable CaP microspheres in inhibiting osteoclast formation, making them a possible alternative to common oral formulations which exhibit low bioavailability, undesirable GI effects, and need frequent administrations [117].

In 2010, an injectable BP-combined CaP matrix was developed with the aim of biologically reinforcing osteoporotic bone incrementing bone fraction and improving bone micro-architecture. The developed system is able to form new bone and to reinforce specific osteoporotic bone site controlling the excessive resorption activity of osteoclasts. Altogether, an injectable carrier based on calcium deficient apatite, which exhibits immediate mechanical properties, could be more suitable than a suspension of calcium deficient apatite granules due to its worse rheological and mechanical properties [118].

Overall, the high potential offered by nanotechnology for the improvement of orthopedic repair and regeneration regards the possibility to ameliorate host bone-implant interaction. However, the safety concern of injectable materials represents the main prerequisite in order to apply nanotechnology for the treatment of osteoporosis.

In addition, a number of new nanomaterials such as injectable hydrogels, NPs or nanocomposites are currently under study by many researchers to optimize their biocompatibility, detecting, and environment-responsive capabilities and drug loading efficiency.

### 3.4. Osteomyelitis

A pilot study was carried out by Rogers-Foy et al. in 1998 with the aim to investigate the possibility of developing a drug delivery system that could both eradicate infection and provide a scaffold for osseous integration. For this reason, they prepared two porous composite systems using HA as carrier for GS to eradicate *Staphylococcus aureus*. Data confirm that the inclusion of GS into ceramic matrix doesn’t affect osteointegration or bone apposition and that this type of system may be beneficial in the treatment of infected osseous sites [119].

Lulu et al. (2018) investigated the efficacy of TOB-loaded CaP beads which can be adopted as a standard care because they enable the localization of supra-MIC levels, difficult to achieve with other substitutes. In fact, this system effectively promoted bone and soft tissue healing and prevented bacterial colonization by *Staphylococcus aureus*. Furthermore, CaP beads show promising anti-staphylococcal activity for the treatment of chronic osteomyelitis in association with TOB, with long period of sustained efficacy [120].

Another tool in the treatment of osteomyelitis is represented by CaPCs. In 2016, Ghosh et al. developed vancomycin-loaded and ciprofloxacin-loaded cements which comprised two synthesized HA powders (HAP): HAP1 and HAP2 with four different weight ratios of HAP1/HAP2: 100/0, 85/15, 50/50 and 0/100. All types of formulated samples inhibited the growth of *Staphylococcus aureus, Escherichia coli*, and *Pseudomonas aeruginosa* with results similar with those obtained with the antibiotic *per se* (ciprofloxacin) or even larger than the vancomycin. The strong antibacterial effect and the biocompatibility with osteoclasts exhibited even by pure cements are noteworthy. This is a highly relevant insight in view of the rising resistance of some pathogens to traditional antibiotics [121].

For the treatment of methicillin-resistant *Staphylococcus aureus* (MRSA) Schnieders et al. (2011) investigated the influence of porosity of CaP composites on the release of vancomycin through the drug-encapsulation into biodegradable microspheres made of PLGA, which were added to CaPCs at different powder to liquid (P/L) ratios with the aim of obtaining cements with different porosities. Results exhibited that the porosity of cement and the vancomycin release profile are both influenced by variations in P/L ratio. In particular, vancomycin-HCl entrapment into PLGA polymer microspheres reduced the influence of the porosity on the cement, while the antibiotic activity of the embedded substance is maintained. Furthermore, after drug entrapment into PLGA matrix, the drug burst is minimized and the drug release is prolonged [122]. Another approach for the eradication of chronic osteomyelitis caused by MRSA was previously tested by Lazarettos et al. (2004). They developed a CaPC enriched with 3% of teicoplanin which showed filler properties of bone defects through newly growing host bone. From the results, it appeared that, due to their spongiform structure, these CaPCs are able to incorporate a double amount of antibiotic in comparison with a solid acrylic material. Furthermore, they exhibit good tissue compatibility, high released drug concentrations and flexibility to the dose and the type of antibiotic that can be loaded into them avoiding reoperation for cement removal [123]. For the prevention of osteomyelitis, Stallmann et al. (2004) developed hLF1-11-loaded and GS-loaded CaPCs and described their efficacy, which was proven to be greater in presence of HLF1-11 [124].

In 2004, Joosten et al. carried out a study in which they investigated the in vitro release of hydroxyapatite cement (HAC) loaded with GS, its mechanical properties and in vivo efficacy. Results suggest that neither the mechanical properties of HAC nor the antibiotic properties of GS were affected when mixed in the concentrations range taken into account. A prolonged release of high levels of GS is obtained with this system which allows a long-term antibacterial effectiveness in situ and in vivo [125].

**Figure 4:**
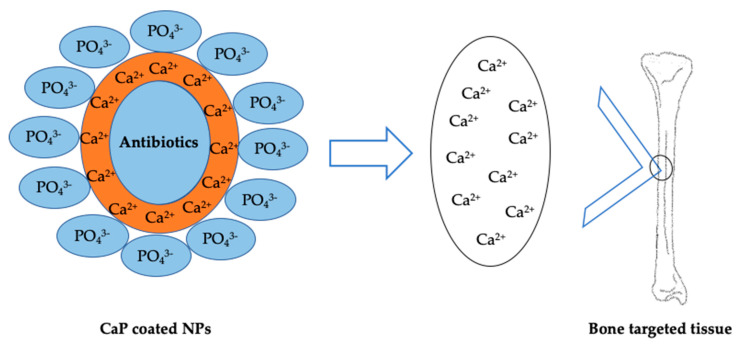
Schematic representation of the mechanism of action of calcium phosphate (CaP) coated nanoparticles (NPs) loaded with antibiotic. Negatively charged NPs could selectively bind Ca^2+^ ions situated on bone surface.

Another tool that has attracted the attention of researchers in last decades is represented by antibiotic loaded particles and CaP-coated NPs which provide a bone-compatible surface (Figure 4).

With this aim, Bastari et al. (2014) developed and investigated the antibacterial activity of PLGA particles coated with CaP. Tests were performed loading two antibiotics, nafcillin and levofloxacin, which are conventional treatments for methicillin-sensitive *Staphylococcus aureus* (nafcillin) and pathogens of chronic osteomyelitis (levofloxacin). A good drug loading efficiency (above 50%) was achieved with both the antibiotics. Furthermore, in vitro tests showed a biphasic release characterized by diffusion and degradation mechanism, confirming the aim of obtaining a sustained drug release up to 4–6 weeks. Inhibition of biofilm formation and its deterioration also occurs [126].

To overcome the burst release of small molecules from HA NPs, Uskoković et al. (2014) coated the drug-containing surface with chitosan, demonstrating that the embedment of 2–10 nm sized HA NPs with chitosan mitigates the burst release of fluorescein (that is bound to HA) and promoted a release time of three weeks. Furthermore, chitosan added to HA could inhibit the proliferation of osteoblastic MC3T3-E1 cell line. However, important drawbacks of these systems concern their lower bacteriostatic efficiency and unviable osteoblastic cell response to the composite material [127].

Previously, in 2013, Uskoković and A. Desai developed agglomerates of NPs of CaP with various monophasic contents. Then, CaP powders were loaded with small molecule drugs showing that the sustained release of purposed systems was dependent on the degradation rate of CaP carriers. Furthermore, through the combination of different CaP phases ratio, drug release profiles could be tailored to the therapeutic occasion [128]. At a later stage, the same authors reported on the effect of the previously synthesized CaPs on bacterial and osteoblastic cell cultures. All CaP powders showed satisfying antibacterial activity against *Staphylococcus aureus*. Moreover, after the adsorption of the antibiotic onto CaP particles, the deleterious effect that the free antibiotic exerted on the osteoblastic cells was reversed, demonstrating that the material developed in the study could be considered for alternative therapeutic approaches to prolonged antibiotic administration in the treatment of osteomyelitis [129].

In 2018, Sampath Kumar et al. developed a dual mode antibiotic delivery system with antibacterial ion substituted calcium deficient HA (CDHA) NPs. Doxycycline was chosen as antibiotic, because it exhibits anti collagenase activity thereby preventing host mediated tissue destruction [130]. Then, ions such as Zn, Ag, and Sr, were incorporated into CDHA, showing an initial high antibacterial activity which can be extended by sustained ion release. Furthermore, AgCDHA and ZnCDHA exhibit high antibacterial activity against *Staphylococcus aureus* and *Escherichia coli*, providing a potent tool to treat incumbent drug resistant infection [131]. Another study carried out by Uskoković et al. was focused on a comparative analysis of two different nanocarriers of clindamycin with and without PLGA coating. Both CaP particles show a positive response about the antibacterial performance of the antibiotic-encapsulated powders against *Sthaphylococcus aureus*, while no cytotoxic or cytostatic behavior resulted after the incubation of free antibiotic or any of the drug carriers with the osteoblastic MC3T3-E1 cell line for up to three weeks. Future perspectives will aim at further enhancing the osteogenic response of osteoblastic cells to these systems as well as the substitution of spherical CaP particles with plate-shaped ones [132].

In 2012, Thanyaphoo et al. produced NPs of silicon (Si)-doped and then, they prepared bioactive ceramics loaded with vancomycin, which is widely applied as a protection against staphylococcal infections. Formulated systems showed a sustained release over a 12-day period that is sufficient for killing *Staphilococcus aureus* growing as a biofilm, without toxicity to bone cells [133].

Considering most of the cited literature data on osteomyelitis topics, the combination of scaffold for tissue regeneration of drugs, and especially with drug delivery systems, stands out as an emerging and successful strategy to ameliorate osteomyelitis treatment, both to improve tissue regeneration and to eradicate microbial infection.

Scaffolds/cements/nanodelivery composites, able to both modulate drug release and perform osseous integration, are a smart and consolidated approach in the treatment of osteomyelitis and represent an interesting therapeutic tool to improve antibiotic efficacy and, in some specific cases, to overcome bacterial resistance.

### 3.5. Bone Regeneration

Currently, studies are mainly focused on the development of 3D composite scaffold and CaPs composites, able to promote tissue regeneration and allow the controlled delivery of therapeutic drugs included growth factors. Biocompatibility, nonimmunogenicity, and inertness to the normal bone healings events are required for the materials used for drug carriers, that usually are composed of natural/synthetic polymers or inorganic minerals. In the literature, different examples are reported, such as gelatin [134], collagen, hyaluronic acid, PEG, PLGA, CaP, HA, and β-TCP, (and also polyphosphates as additives [135]), but in the case of natural polymers, there are some disadvantages connected to their use, like immunogenicity, rapid degradability, and batch-to-batch variations. On the other hand, synthetic polymers offers the possibility to be easily modified and processed to obtain the suitable properties via the variation of their molecular weights, functional groups and/or structure [136]. Currently, CaPs, and in particular HA and TCP, are widely used in bone tissue engineering as bone substitutes and drug carriers, for their biocompatibility, osteogenic properties and their similarities to bone mineral with which they are able to form strong bonds [137].

In literature a great amount of studies reports the use of 3D porous scaffolds with drug delivery capability for bone regeneration [138]. In 2018 Yao et al. developed mesoporous silica NPs (MSNPs) incorporated in gelatin nanofibrous dual-drug delivery system to obtain a controlled release of macromolecules like BMP-2 and deferoxamine. At the same time, this system, thanks to its excellent bioactivity, could be useful to reduce high cytotoxicity, off-target effects and the short half-life of deferoxamine. Results showed that the formulated scaffold could actually provide a controlled dual-release at distinct release rate of both drugs loaded with the maintenance of their angiogenic and osteogenic abilities [139].

In 2017, Song et al. developed an injectable cell-encapsulating alginate-fibrin hydrogel microfiber-CaPCs tissue-engineered system. It resulted that the injectable construct is able to regenerate new bone in vivo three-fold more than the control, with a quick degradation of the microfibers and release of the cells. In addition, the system maintains its viability and osteogenic potential which make it interesting in bone regeneration for several applications, like dental, craniofacial, and orthopedic surgeries [140].

In the same year, Frasca et al. carried out a study in which the bone healing process induced with a porous pullulan/dextran-based hydrogel scaffold and a commercial HA/TCP ceramic (also in association with mesenchymal stem cells) were compared, showing that the first system could be considered as an alternative or complementary tool for its osteogenic properties, rapid resorption, and capability to deliver growth factors [141].

Wang et al. (2016) encapsulated stem cells of different origins in alginate-fibrin hydrogel fibers, which were then mixed with CaPCs paste to form an injectable system with fast setting time and high porosity, and investigated which type of stem cells would be superior in osteogenesis. Results reveal that formulated systems are able to enhance bone regeneration and that they could be used for dental and orthopedic applications [142]. In 2015, Nazemi et al. studied the effect of adding PLGA NPs on a chitosan-bioactive glass (chitosan-BG) scaffold revealing that the presence of NPs could increase the mechanical strength of the scaffold, reducing its swelling behavior. Moreover, the addition of NPs makes the system suitable to be implanted as a controlled-release platform of model drugs [143]. The aim of the study carried out by Zhang et al. (2015) was to develop and to evaluate CaP composite scaffolds containing simvastatin-loaded PLGA microspheres. Data analysis exhibits a certain biocompatibility and osteogenesis in vitro and in vivo, improving the efficiency of new bone formation [144]. Another two important works concerned the study of in vitro and in vivo behavior of PLGA/CaPCs scaffold with a lamellar pore structure [145] and biphasic CaP scaffold loaded with ALN [146,147], which demonstrated biocompatibility and effective osteogenesis, characteristics which make them potential tools for bone tissue engineering in orthopedic and dental fields.

Hydrogel-based systems have several advantages such as the ability to provide a hydrophilic environment suitable for new bone growth, the possibility to be tailored for a better geometry, degradation rate and release profile [148]. Hydrogels can be produced with one or more-step methods like polymerization or the synthesis of polymer molecules. Important factors which may influence many of the properties of the hydrogel are the type and the degree of crosslinking [149]. A wide variety of hydrogel formulations can be developed via different preparation methods. In literature, for example, a recent study by Tangprasert et al. (2017) involved the development of a mimicked extracellular matrix composed of gelatin/chitosan/CaP compounds in different ratio with the aim of obtaining an ex-vivo model for the evaluation of tissue formation. Indeed, the results showed that formulated systems are similar to extracellular matrix of bone, as well as able to enhance cell viability and proliferation [150].

To obtain an advantageous biomaterial for bone tissue engineering, Yu et al. (2016) drew up an easy method to synthetize graphene hydrogels by self-assembling of graphene oxide, HA NPs and chitosan into a 3D hydrogel. The resulting composite exhibits high mechanical strength and capacity to binding HA, meeting the requirement of bone materials [151]. As an alternative to the use of autologous tissues, two studies attracted our attention, one carried out by Weinand et al. (2006) and the second by Schröder et al. in 2015. In the former case, researchers combined mesenchymal stem cells suspended in hydrogels and 3D β-TCP scaffolds, obtaining a compound which can support bone formation in vitro (especially in presence of collagen I hydrogel) [152]. In the latter case, Schröder et al. demonstrated the promising characteristics of biodegradable PEG hydrogel loaded with vaterite (a polymorph of CaCO_3_) NPs, stable enough to obtain a potential ion buffer for bone regeneration but also capable of allowing the conversion of CaCO_3_ into HA. Evidence revealed the potential bone targeting capability of this type of systems as well as a certain biodegradability. In this way the hydrogel would not stay persistently in the body but could serve as a scaffold for the regeneration of new bone tissue. Moreover, they don’t show any inflammatory potential [153]. A 2014 study by Douglas et al. provided for the mineralization of gellan gum hydrogels by the incorporation of alkaline phosphatase in media containing calcium and/or magnesium glycerophosphate at different ratios. Results demonstrated that calcium is incorporated better than magnesium and that the rigidity of the compound is related to the mineralization medium. Furthermore, when magnesium is incorporated into mineral, an increase in osteoblast attachment and proliferation can be observed [154].

Hydrogels could be used also as bone-filling materials. In particular, Nonoyama et al. in 2012 prepared peptide hydrogels though the mineralization of ACP and HA, demonstrating that the above mentioned systems have a viscoelastic strength sufficient to be injected and that the calcium ions are at the same time a source of minerals and a reinforcing agent which can increase the mechanical strength of hydrogels [155]. At the end, another noteworthy study for our purpose is that of Leeuwenburgh et al. (2007) in which the researchers developed composite hydrogels in which CaP nanocrystals were precipitated in presence of dissolved oligo(poly(ethylene glycol)fumarate) macromers, demonstrating that, in this way, the swelling behavior of this compound can be reduced with an increment of its cross-linking [156].

Another tool which possesses some advantages in the goal of bone regeneration medicine is represented by NPs. For this purpose, Qu et al. (2016) drew up a multilayered coating made of PLGA NPs, heparin and chitosan for the delivery of dexamethasone and rhBMP-2. It seemed that PLGA NPs could be transported across the cell membrane improving the intracellular delivery of dexamethasone through the internalization inside cells. In this way researches demonstrated the possibility of a dual sustained release of dexamethasone and rhBMP-2 with a coating that could be combined with others biomaterials enhancing their osteogenic properties [157]. Another study (2016) in the literature involved the use of lithium modified BG NPs for the co-delivery of lithium ions and other drugs as a possible treatment in a first instance for patients with osteomyelitis, osteoporosis, and bone cancer but, later, also for bone engineering. Researchers obtained a prolonged release of loaded drug for 32 days with a release profile depending on the concentration of the drugs loaded during the preparation of the system [158].

Recently, microparticulate systems represent a valid tool for the treatment of defective bones (e.g., implantation and maxillofacial treatment), thus different research involved the study of various systems eventually loaded with bioactive drugs and/or molecules. For example, in 2011, Park et al. developed microspheres composed of CaPCs incorporating biological proteins which can be released in a sustainable way over a month. Adhesion and proliferation of the cells cultured upon microspheres was observed, making these systems potentially useful as 3D matrix [159]. With the same purpose, Ribeiro et al. (2004) prepared CaP-alginate and HA-alginate microspheres loaded with enzymes [160].

**Figure 5:**
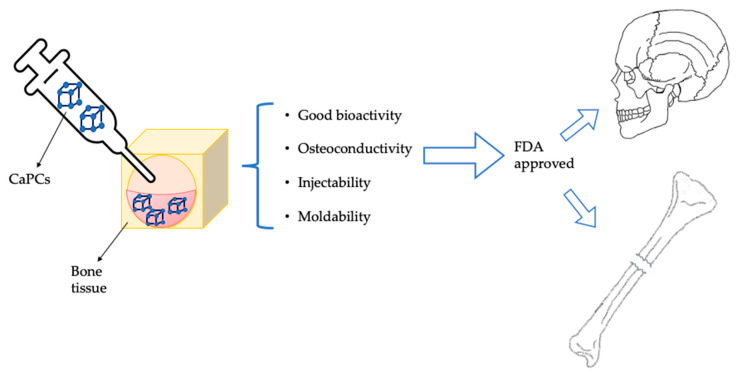
Schematic representation of the advantageous characteristics of bone injected calcium phosphate cements (CaPCs).

On the other hand, CaPCs could represent a promising alternative for bone disease due to their good bioactivity, osteoconductivity, injectability, and moldability. As mentioned above, they were approved by FDA for the treatment of craniofacial defects and bone fractures (Figure 5) [161].

An interesting study found in literature (2016) reports the development of nanostrontium HA-enhanced CaPCs and simvastatin-loaded PLGA microspheres plus CaPCs composite for increasing bone formation. In fact, some studies confirm that strontium can stimulate osteoblast differentiation and inhibit osteoclast activity. Results suggest that the strontium-CaPCs exhibit good biocompatibility and induce higher bone regeneration, while strontium-CaPCs containing simvastatin-loaded PLGA microspheres show higher levels of osteogenic differentiation. It appears that, in presence of nanostrontium there is an improvement of the chemophysical properties of CaPCs with a reduction of disturbances factor for the tissue like inflammation. Furthermore, in the presence of PLGA microspheres loaded with simvastatin, better results were obtained in terms of osteogenesis compared to strontium-CaPCs alone [162].

Finally, a good alternative is represented by HA-composites with reduced crystal dimensions in order to obtain a hard tissue replacement. In 2006, Boanini et al., based on the evidence that ASP and GLU play a role in bone cell signaling, explore the possibility to develop HA-ASP and HA-GLU crystal. Results show that HA-ASP and HA-GLU nanocrystals are able to help the growth of MG63 cells in vitro and promote their differentiation, by promoting osteoblast proliferation and metabolism/differentiation activation [163]. More recently, in line with these findings, HA NPs loaded with both Mg^2+^ and Zn^2+^ ions were used to coat BG-based scaffolds of 45S5 composition and tested as materials for bone tissue engineering applications [164]. These materials exhibited higher/faster bioactivity and an increase of proliferation of MG-63 cells than uncoated scaffolds. In agreement with these results, SEM images confirmed increased interaction between coated scaffolds and cells. Altogether, the obtained results suggest that nanocrystalline Mg-Zn dopped-HA coatings may enhance the biological performance of standard scaffolds of 45S5 BG composition.

From literature data on the topic, it emerges that CaPs such as HA play an important role due to their similarity to bone mineral phase and to their osteoinductive properties.

Among the many cited systems in this paragraph, the authors think that CaP nanoparticulate systems represent the strategy of the future, as they have been successfully used for the delivery of several therapeutic factors for bone repair, comprised gene delivery.

Further efforts should be made to fully understand and mimic the natural healing process of bones, a mandatory requisite to develop the ideal CaP-based nanocomposite system.

## 4. Summary of the Commonest Drug Delivery Systems for Bone Diseases

A concise summary of the bone drug delivery studies examined in this review is reported in Table 9. For this purpose, each drug delivery system is linked to its application field, main advantages and disadvantages, and relative references.

**Figure 6:**
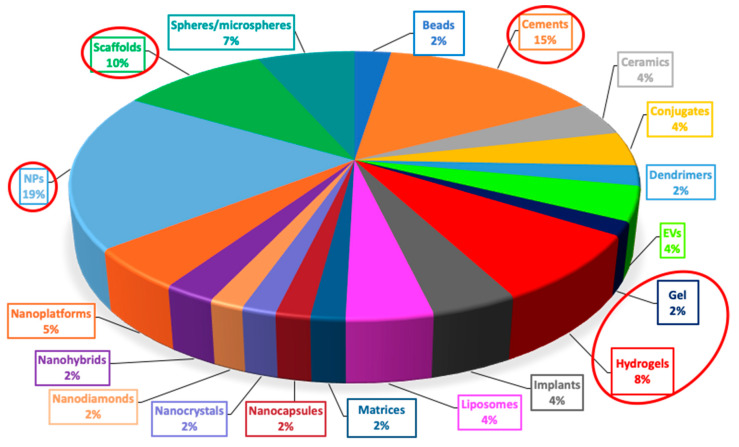
Schematic diagram showing citing frequency of each bone drug delivery approach as percentage of total number of the studies cited in this review.

Different types of drug delivery systems such as NPs, cements, scaffold and gel/hydrogels have been recently exploited for the treatment of bone diseases. As represented in the diagram below (Figure 6), among all the studies cited in this review, almost 54% consists in the above-mentioned drug delivery systems (19%, 15%, 10%, and 10%, respectively). Such a trend is probably due to their peculiarities, e.g., bioactivity, osteoconductivity and biocompatibility. Furthermore, several studies demonstrated that a controlled and prolonged drug release could be obtained with these systems which could also be tailored (by coating or changing of surface charge) in order to improve stability and receptor binding, or to optimize the therapeutic effects.

Other drug delivery systems, like spheres and microspheres, nanoplatforms, liposomes, EVs, implants, conjugates, and ceramics, cover a minor range (4–7% of the total studies), showing promising properties as cargo of cellular content, hydrophilic/hydrophobic drugs, and biomolecules (e.g., DNA fragments, peptides, proteins).

## 5. Conclusions

Conventional therapies in the treatment of bone disease are still widely used although they exhibit several disadvantages such as lack of patient compliance due to adverse reactions of administered drugs and to the invasiveness and the low selectivity of the treatment.

In the last decades, nanotechnology has come to represent an attractive choice in order to overcome these problems, mainly owing to the versatility of nanosystems that, if properly functionalized, can selectively reach the site of bone disease and prolong drug release. A wide range of nanoscale matrices, like polymers and ceramics, are the most used materials to produce these systems which can be further improved by tailoring surface properties, for example with CaP, that exhibit characteristics similar to bone components. A considerable part of these innovative delivery systems has been studied at a preclinical level, indicating only a moderate positive effect for some of them, such that further investigations are certainly required for clinical trials and following commercial purposes.

Evidently, not all drug delivery strategies are equally promising. Overall, compared to other platforms, cements seem to provide more opportunities being the most versatile approach already widely employed from OS to osteomyelitis and bone regeneration. In parallel, nanotechnologies have demonstrated, mainly in preclinical trials, significant benefit in both targeting and prolonging drug release. For these reasons, we believe that in the near future therapies combining bone cements, especially HA and TCP, with drug loaded nanocarriers can offer a successful approach for the treatment of bone diseases.

Undoubtedly, ongoing research and future perspectives will provide for the further improvement of the characteristics and the selectivity of these systems.

Technologies such as quality-by-design, process analytical techniques and microfluidics could significantly accelerate the translation of nanomedicines. Nevertheless, this kind of approach requires further learning and an adequate regulatory background. Overall, to achieve an efficient clinical translation, collaboration among academia, industry, and regulatory bodies is required to ensure safe and effective nanomedicine products.

## Figures and Tables

**Table 1:** Bone metastases characteristics [15].

Types	Pathophysiology	Primary Cancer Sites
Osteolytic	Destruction of normal bone by ostoclasts	Multiple myeloma, melanoma, non-small cell lung cancer, non-Hodgkin lymphoma, thyroid cancer or Langerhans-cell histiocytosis, breast cancer, renal cell carcinoma
Osteoblastic	Deposition of new bone by osteoblasts	Prostate cancer, small cell lung cancer, Hodgkin lymphoma or medulloblastoma
Mixed	Osteolytic and osteoblastic lesions or both types present in the same lesion	Breast cancer, gastrointestinal (GI) cancer and squamous cancer

**Table 2:** Common drugs and therapeutic techniques for the treatment of bone metastases.

Agent	Mechanism of Action	Side Effects
Bisphosponates (BPs)	Inhibition of bone demineralization by the interruption of enhanced osteolysis and tumor growth [16]	Fever, arthralgia, myalgia, anemia, nausea and peripheral edema. Osteonecrosis of the jaw (rare, difficult to establish casative evidence) [17]
Tetracyclines	Inhibition of matrix metalloproteinases (MMPs) involved in bone metastasis [17]	Dose-limiting toxicity e.g., fatigue and nausea, development of resistance [18] Generally well tolerated in adults
Denosumab	Inhibition of RANKL which prevents the development of osteoclasts	Similar to those exhibited by BPs, but reversible after treatment interruption [15]
Cabozantinib	Inhibition of vascular endothelial growth factor receptor-2 (VEGFR2), MET, KIT and mutationally activated RET [15]	Fatigue, diarrhea and palmar-plantar erythrodysesthesia syndrome [19]
Radionuclide therapy	Systemic administration of radioisotopes, but the mechanism of pain relief is uncertain [20]	Myelosuppression and pain flare [15]
Ablation	Use of chemical agents (ethanol, acetic acid) or local deposition of some form of energy (e.g., for radiofrequency and cryoablation) to destroy tumor cells [21]	Neurologic injuries, neuropathic pain and infection in the treatment area (especially for radiofrequency and cryoablation) [22]

**Table 3:** Classification of Osteosarcoma (OS).

Types	Subtypes	Frequency
Intramedullary	Conventional (osteoblastic/chondroblastic/fibroblastic)	80%
	Telangiectatic	<4%
	Low-grade	1–2%
	Small cell	1.5%
Cortex-associated	Parosteal	1–6%
	Periosteal	1–2%
	High-grade surface	<1%

**Table 4:** Chemotherapeutic agent used in the treatment of OS [25].

Agent	Mechanism of Action	Side Effects
DOX	Intercalation at points of local uncoiling of the DNA double helix and inhibition of DNA/RNA synthesis	Cardiomyopathy, emesis, alopecia, mucositis, myelosuppression
Cisplatin	Formation of DNA cross-links with inhibition of the synthesis of the tumor DNA and denaturation of DNA double helix	Acute/chronic renal failure, ototoxicity, emesis, myelosuppression, alopecia, hypomagnesemia
Ifosfamide, with mesna	Cross-linking of DNA strands with inhibition of DNA/protein synthesis	Hemorrhagic cystitis, renal failure, myelosuppression, alopecia, emesis, encephalopathy
High-dose methotrexate (MTX), with leucovorin calcium rescue	Inhibition of purine/thymidylic acid synthesis by binding dihydrofolate reductase	Renal failure, mucositis, myelosuppression, nervous system effects (rare)

**Table 5:** Characteristics of the two main classes of antiresorptives [28].

Drug	Advantages	Disadvantages
N-BPs	Bone selectivity, inexpensive, longtime use, apoptosis of osteoclasts inductors (but don’t eliminate them)	Effects on renal functions, atypical femoral fractures (osteonecrosis of the jaw), GI adverse effect, acute phase reactions
Denosumab	No effect on renal function, low frequency of administration, no bone accumulation, reversible effects	No bone selectivity, atypical femoral fractures, increased risk of infections, parenteral administration, reduction in bone formation, rebound bone resorption

**Table 6:** Characteristics of newer antiresoprtives and selective estrogen receptor modulators (SERMs) [28].

Class	Drug	Characteristics
Cathepsin K inhibitors	Odanacatib	Ability to decrease the rate of bone resorption, preserving other osteoclasts functions Orally active, weekly administered Phase III fracture clinical trial
SERMs	Raloxifene	Ability to decrease the risk of vertebral fractures by 30% First FDA approved SERM
	Bazedoxifene in association with TSECs	Treatment of moderate/severe hot flashes associated with menopause Approved by FDA in 2013 and in the European Union in 2009 (to be used alone)
	Lasofoxifene	Double event rate for venous thromboembolic events

**Table 7:** Characteristics and disadvantages of sclerostin inhibitors [28].

Drug	Origin	Characteristics	Disadvantages
Romosozumab	Humanized mouse monoclonal antibody	Increment of lumbar spine and hip bone mass density during the first year of treatment Phase II studies	Benign safety profile need to be confirmed Probable development of antibodies against them Probable risk of vascular calcification Parenteral administration
Blosozumab	Humanized mouse monoclonal antibody	Under investigation	

**Table 8:** Traditional materials used for bone repair [33].

Material	Type of material	Advantages	Disadvantages
Bone	Autologous bone	Biocompatible with high bone fusion rate	Limited sources
	Allogenic bone	Relatively high bone fusion rate	Immune rejection
	Heterologous bone	Wide variety of sources	Severe immune rejection and poor bone formation
Bone cement	Non-bioactive bone cement	Easy fit, good hardening properties	Poor biocompatibility, non-osteoinductive, non-osteoconductive
	Bioactive bone cement	High strength, stability and bone induction activity	Insufficient mechanical properties, expensive
Metal	Stainless steel	Easy processing, inexpensive	High stiffness, poor biocompatibility
	Titanium alloy	Biocompatible, corrosion resistance	Poor wear resistance
	Cobalt chromium alloy	Biocompatible, high corrosion resistance	Low ductility
Ceramic	Aluminium oxide	Inertness, high corrosion resistance	Possible local stress
	Apatite-wollas-tonite glass ceramic	Good biological activity	Brittleness, poor flexibility
Polymer	Poly(lactic-co-glycolic) acid (PLGA)	Biocompatible, degradable	Possible disruption
	PMMA	Corrosion resistance, easy fit	Poor biocompatible
	Chitosan	High degradable and biocompatible, porous structure, good mechanical properties	Non-osteoinductive, inadequate bone formation ability, low solubility
	Alginate	Easy to manipulate, non-toxic, biodegradable, less expensive	Low mechanical stability

**Table 9:** Summary of the studies on bone drug delivery examined in this review.

System	Applications	Advantages/Disadvantages	References
Beads	OS	Carrier of low molecular weight drugs Best results with alginate/chitosan association Time-dependent, but burst and inconsistent release profile Non-biodegradable, need to be surgical removed	[44,45,120]
Cements	Bone regeneration Bone metastases Osteoporosis Osteomyelitis OS	Ability to harden in vivo and to form bond with bone, low temperature setting reaction, excellent bioactivity, osteoconductivity Low strength, lack of full injectability, slow resorption rate, limited amount of incorporated drug, heterogeneity of drug repartition	[50,51,83,94,95,100,101,102,103,104,105,121,122,123,124,125,161,162]
Ceramics	Osteomyelitis	Simple preparation, modifiable size and structure, desirable stability under physiological conditions, low toxicity, good biocompatibility High cost, low encapsulation, uncontrollable dose	[2,52,53,133,137]
Conjugates	Bone metastases Osteoporosis	Good aqueous solubility, stability and controlled delivery Better oral bioavailability, tumor targeting, reduced toxicity for lipid-drug conjugates	[48,49,77,79,80]
Dendrimers	Bone metastases	Drug/oligonucleotides easily encapsulated Monodisperse size, good water solubility Modifiable surface Markable cytotoxicity for cationic surface end groups	[47,73,75]
EVs	OS	Cargo of cellular content, drugs and biomolecules	[38,39,84,85,86]
Gel	Osteoporosis	Controlled and continuous drug release High therapeutic effects, capable of auto-forming in situ	[58,116]
Hydrogels	Bone regeneration	Sol-gel transitions due to body temperature or pH variations Possibility to be tailored for a better geometry, degradation rate and release profile Development of a hydrophilic environment suitable for new bone growth, fast setting time Suitable for dental and orthopedic applications	[142,148,149,150,151,152,153,154,155,156]
Implants	Osteoporosis	Biocompatibility, prolonged drug release, tailored biodegradation kinetics Suitable for cardiovascular, neuroprosthetic and orthopedic purpose Required surgical procedures with some side effects like bleeding, infections or nerve injuries (for dental implants) High production costs and the possible presence of degradation fragments which impair new grown bone	[62,106,107,108,119]
Liposomes	Bone metastases OS	Good biocompatibility and loading properties Possible prolonged/targeted drug delivery	[40,71,72,90,91]
Matrices	Osteoporosis	Improved patient compliance, treatment efficiency, reduced costs, possibility to reduce drug administrated Probability of dose dumping, poor in vitro/in vivo correlations	[59,118]
Nanocapsules	Osteoporosis	Protective coating which delays compounds release, high reproducibility, broad range of application	[60,115]
Nanocrystals	Bone regeneration	Suitable for intravenously injection, slowly blood dissolution, improved biodistribution Opsonization could prolong circulating time, promote tumor accumulation, improve targeting efficiency	[55,163]
Nanodiamonds	Osteoporosis	Large surface area, easiness surface functionalization, suitable as photoluminescent probes Negligible cytotoxicity, high biocompatibility	[54,114]
Nanohybrids	OS	Capable to incapsulate drug with low water solubility, possible tool for non-invasive tumor targeting Good biocompatibility and biodegradability	[56,57,97]
Nanoplatforms	Bone metastases Osteomyelitis	Biocompatible and biodegradable Tunable size, changeable membrane permeability, possible bound with targeting ligand, capable to load hydrophilic/lipophilic drugs Responsive to pH, ionic strength, temperature	[30,31,64,81,82,143]
NPs	Bone regeneration Bone metastases Osteoporosis Osteomyelitis OS	High soluble, bioavailable Possible surface coating or functionalization to improve stability and bone targeting	[32,43,46,65,66,67,68,69,70,87,88,89,110,111,126,127,128,129,130,131,132,157,158]
Scaffolds	Bone regeneration Osteomyelitis	Biocompatible templates, promote attachment and growth of bone cells, induce osteogenesis Possible drug carriers	[61,63,96,138,139,140,141,144,145,146,147,164]
Spheres and microspheres	Bone regeneration Osteoporosis Osteomyelitis OS	Reduced side effects and improved efficiency of cytotoxic anticancer drugs Alternative routes: ophthalmic, topical, nasal, intramuscular Good choice for low/high molecular weight drugs, DNA fragments, peptides, proteins	[41,42,92,93,112,117,159,160]

## Abbreviations

ACP	Amorphous calcium phosphate
ALN	Alendronate
ASP	Aspartic acid
Au@MSNPs	Gold nanorods enclosed inside mesoporous silica nanoparticles
BG	Bioactive glass
BMP	Bone morphogenetic protein
Bip	3,5-di(ethylamino-2,2-bisphosphono) benzoic acid
BPs	Bisphosphonates
BSA	Bovine serum albumin
CA	Citric acid
CaP	Calcium phosphate
CaPCs	Calcium phosphate cements
CaPSC	Calcium phosphate silicate cements
CDHA	Calcium deficient hydroxyapatite
CPNs	Coordination polymer nanoparticles
CPPA	Calcium phosphate-phosphorylated adenosine
CSC	Calcium silicate cements
DOX	Doxorubicin
DSP	Diamminedichlorosuccinate-platinum
DTPA	Diethylenetriaminepentaacetic acid
DTX	Docetaxel
EMA	European Medicines Agency
EVs	Extracellular vesicles
FDA	Food and Drug Administration
FGF	Fibroblast growth factor
GI	Gastrointestinal
GLU	Glutamic acid
GS	Gentamicin sulfate
HA	Hydroxyapatite
HAC	Hydroxyapatite cement
HAP	Hydroxyapatite powder
IGF	Insulin-like growth factor
IL	Interleukin
I.V.	Intravenous
MA	Monoethyl adipate
MMPs	Matrix metalloproteinases
MRI	Magnetic resonance
MRSA	Methicillin-resistant *Staphylococcus aureus*
MSCs	Mesenchymal stromal cells
MSNPs	Mesoporous silica nanoparticles
MTP-PE	Muramyl tripeptide phosphatidyl ethanolamine
MTX	Methotrexate
N-BPs	Nitrogen-containing bisphosphonates
NIR	Near infrared
NPs	Nanoparticles
OCP	Octacalcium phosphate
OS	Osteosarcoma
P/L	Power to liquid
PAMAM	Polyamidoamine
PDGF	Platelet-derived growth factor
PEG	Polyethylene glycol
PEP_n_ Na	Poly(ethylene sodium phosphate)
PL	Plumbagin
PLA	Polylactic acid
PLGA	Poly(lactic-co-glycolic) acid
PMMA	Poly(methyl methacrylate)
PNs	Polymeric nanoparticles
PTH	Parathyroid hormone
PTT	Photothermal therapy
PTX	Paclitaxel
RA	Risedronate
RANKL	Nuclear factor kappa-B ligand
ROS	Reactive oxygen species
SERMs	Selective estrogen receptor modulators
SPIONs	Superparamagnetic iron oxide nanoparticles
TCP	Tricalcium phosphate
TEs	Trace elements
TGF-β	Transforming growth factor-β
TNF	Tumor necrosis factor
TOB	Tobramycin
TSECs	Tissue-selective estrogen complexes
VEGF	Vascular endothelial growth factor
VEGFR	Vascular endothelial growth factor receptor
W/O	Water in oil
ZOL	Zoledronate
